# Extract from a Monograph on Local Anæsthesia in Dentistry and Minor Surgery

**Published:** 1887-05

**Authors:** George. Viau, L. Turnbull

**Affiliations:** Philadelphia.


ARTICLE V.

### EXTRACT FROM A MONOGRAPH ON LOCAL ANÆSTHESIA IN DENTISTRY AND MINOR SURGERY, BY PROF. GEORGE. VIAU.*

TRANSLATED BY L. TURNBULL, M. D., PHILADELPHIA.

Dr Aubeau employs in his experiments a solution of cocaine, 5 per cent., that is to say, that in every Pravaz syringe containing a gramme of distilled water he dissolves only 5 centigrammes of cocaine, an insufficient quantity for cases where the roots of the teeth are firmly set in the alveolar process.

Mr. Telschow injects 10 centigrammes of cocaine, evidently too powerful a dose, as it produces general trouble in a number of cases.

Prof. Viau combines another local anæsthetic with a small quantity of cocaine, 5 centigrammes, which unites in acting with the cocaine without affecting the general health. This second substance is pure crystallized phenic acid. This acid neutralizes the effect of the solution of cocaine, and has been used with success. Mr. Telschow weakens his solution of cocaine with the phenic acid to neutralize it. The author finds an important agent in the acid, the anæsthetic properties of the pure or concentrated acid being known to all who have used it. It is the anæsthetic, *par excellence*, of intelligent dentistry. The author now describes his two modes of administering anæsthetics:

1. Anæsthesia by the aid of sub-mucous injections with a mixture of cocaine and phenic acid.

2. Anæsthesia by the aid of sub-mucous injections with a solution simply of phenic acid.

Local anæsthesia obtained by the aid of cocaine and

* Officer of the Academy, Professor of the Dental School and Hospital, Paris; Secretary General Association of Dentists in France; Member of the Council for the Direction of the Dental School.

phenic acid mixed.

Before beginning to describe this process, I should relate an experiment that I made for the purpose of investigating the properties of a solution of pure cocaine (alkaloid) in the pure crystallized phenic acid. In mixing one part of phenic acid and two parts of cocaine, heating it very slightly, a product of a syrupy quality is obtained, which retains the color of the pure phenic acid, but dissolved; that is to say, rosy; and the odor weakened by the acid. The taste on the contrary is decidedly modified. The phenic acid has lost its causticity and its peculiar taste. In using this product upon the gums or tongue a sensation of warmth is perceived with the taste of the weakened phenic acid, allowing the bitterness of the cocaine to penetrate. At the end of several seconds the taste and the odor of the phenic acid disappear completely, at the same time that the sensation of heat is perceived at the moment of contact, the taste of the cocaine alone remains, with complete insensibility to pain.

Have I obtained in this manner a phenate of cocaine, or have I produced a simple solution? I leave to those more competent the solution of this question.

However it may be, I have a product which fully answers my requirements. Below is a succinct description of my operation procedure, thus:

1. The filtered solution:

| | |
|---|---|
| Crystallized phenic acid, - - - | 2 gr. |
| Distilled water, - - - - | 100 gr. |

2. Package of hydrochlorate of cocaine. A syringe of Pravaz, which contains a gramme of water, in which I made a slight modification by adding to the opposite side of the canula a shoulder piece, which enables me to hold it with greater ease and strength, with the index and middle finger, whilst I press the stem of the piston with the thumb. I have also invented sharp canulas of different lengths, which are absolutely necessary, in difficult cases of contraction of the jaws, to reach the wisdom teeth. I

dissolve, when required, 5 centigrammes (or 1 grain) of cocaine in 50 centigrammes (10 drops) of phenic solution and inject slowly half of the mixture into the "labial face," half into the lingual or palatine—at a point situated between the neck of the tooth and the presumed place of the extremity of the root, to within 2 or 3 millimetres of that extremity. I am careful to hold a finger, of the left hand, upon the puncture, to prevent the liquid from flowing backwards. The patient must then wash out the mouth with fresh water. At the end of three minutes the soft parts are completely insensible. A very deep puncture is no longer felt. Between the fifth and sixth minute I operate. This process has always given me complete results, as regards anæsthesia. As to general troubles I have never tried them. Sex and age appear to have no influence on the anæsthethic results. I have operated upon 30 males and 56 females—total, 86 subjects. It will be seen in the table of observations that several subjects have received the anæsthetic twice at the same sitting, without having been otherwise affected. These patients have, however, absorbed in a lapse of some minutes, 10 centigrammes of cocaine in a gramme of phenic solution. Other patients have undergone, with several hours' interval, two or three operations, always with success. I have also observed that the patients upon whom I operate for the second or third time have lost all fear. In submitting thus to the operation, it is evident that this facilitates many anæsthetic injections—a work requiring great precision. Observations show that the half of the ordinary dose of anæsthetic—that is to say, about 2 centigrammes, ½ of cocaine in 25 centigrammes of phenic solution—has sufficed to produce anæsthesia.

In conclusion, I think I will be able to diminish hereafter the quantity of cocaine, and to inject less liquid under the gums; altogether, obtaining a complete local anæsthesia for the extraction of teeth.

REMARKS BY TRANSLATOR.

In a recent lecture, delivered by invitation before the

Philadelphia Dental College, on Anæsthetics, we dwelt at considerable length on the great value of a true and successful local anæsthetic, recommending cocaine, and yet cautioning the use of a stronger solution in the eye than 1 per cent.; and for the extraction of teeth, 4 or 5 per cent., but in the ear and throat, a much stronger solution. Notwithstanding all precaution being taken, unsatisfactory cases will arise; patients of nervous or hysterical natures are subject to nausea and trembling, followed by depression, with loss of muscular power; and even comatose cases requiring powerful restoratives, while some state that no pain whatever is felt.

All interested are advised to read, with care, the unfortunate case on pages 49, 50 and 51 of the "Appendix to Manual of Anæsthetics."

We also stated that up to this time, December, 1886, no well authenticated case of death from cocaine has been reported, which was a subject of congratulation.

One sad death has, however, occured in connection with cocaine; whether caused by it or not, remains a mystery, as the unfortunate operator destroyed himself. An extract from the *London Lancet* of an article taken from *Semaine Medicale*, gives some particulars of the fatal accident which led to the suicide of Professor Kolommine. The Professor had decided to perform an operation for the removal of tubercle from the rectum of a female patient, and inquired of his colleague, M. Louschinsky, what dose of cocaine he might administer. The answer was, that the maximum was 2 grains. Upon consulting special treatises, however, M. Kolommine found that 30 cases of anæsthesia were recorded in which the quantity given had varied between 6 and 96 grains. In a similar operation to that he proposed to perform, a French surgeon had given 48 grains. In consequence of these references, it was decided to employ 24 grains, which were introduced into the rectum by instalments. About half an hour after the operation, the first symptoms of poisoning appeared, and notwithstanding the administration of nitrate of amyl, hypodermic injections of ether, oxygen, and artificial respiration, etc., the patient succumbed.

Hypodermic injections of morphia do not seem to have been tried, and I do not know whether they have been recommended in cases absolutely similar to this.

I may mention, however, that I have seen symptoms of cocaine poisoning, in subjects of morphia habit, relieved instantly by a hypodermic injection of morphia, and in one case 5 grains of cocaine had been taken hypodermically, within ten minutes; and I have frequently seen the same antagonistic effect produced by a small quantity of cocaine, is an overdose of morphia. It is also to be regretted that strychnia was not tried in this case, for the experiments of Dr. Bignon have shown that cocaine is its physiological antidote, and it is quite possible that the antagonism may be reciprocal. Dogs poisoned by strychnia to the extent of 1 milligramme (a fiftieth of a grain) per pound, can always be saved by keeping up cocaine intoxication, until the elimination of the strychnia. See also the suggestion of Dr. Briggs, of New York, of the use of cocaine in tetanus and strychnia poisoning. (*Journal of the American Medical Association*, January 17, 1885, page 37; also Turnbull Appendix to Manual of Anæsthetics, page 62, last edition, P. Blakiston, Son & Co., Philadelphia.)

We were highly delighted to find from the pamphlet of Professor Viau that he had been able to modify the hydrochtorate of cocaine in solution with pure phenol, so as to prevent, in 87 cases, any unpleasant results from its use in the extraction of teeth. The solution was prepared as directed by the Paris Dental Surgeon, and before a class of about 200 students. The mixture was injected into the jaw, in a case of diseased antrum in a young lady. She was then operated upon by Dr. Garretson, who removed the diseased bone with the dental engine. She bore the operation with great equanimity, and with so little pain as to be unnoticed by the students; and when asked at the completion of the operation, she stated that she experienced some slight pain. The case was an interesting one, in its freedom from excitement and haste, and the quiet way in which she would rise, expectorate the blood, and be cleansed from the horrible disfigurement, avoiding the great risk produced by profound anæsthesia, which is so nigh unto death, required in such an important operation.

A little girl was also operated upon, for the extraction of teeth; she cried when the hypodermic syringe pricked her, yet in seven minutes the very much decayed tooth was extracted without the slightest evidence of pain. We think this combination has a wonderful future.—*Dental Advertiser*.

